# Enhanced thin-film transistor driven high-aperture in-plane switching liquid crystal displays without common line and black matrix

**DOI:** 10.1038/s41598-021-90924-x

**Published:** 2021-06-01

**Authors:** Masahiko Ando, Makoto Yoneya

**Affiliations:** 1 R&D Group, Hitachi, Ltd., 1-280 Higashi-Koigakubo, Kokubunji, Tokyo 185-8601 Japan; 2 Advanced Industrial Science and Technology, 1-1-1 Umezono, Tsukuba, 305-8568 Japan

**Keywords:** Engineering, Nanoscience and technology, Physics

## Abstract

We developed active-matrix in-plane switching liquid crystal displays (IPS-LCDs) with a new vertical structure composed of thin-film transistors (TFTs) that have an aperture ratio of 60% to reduce energy consumption. The novel TFT has a channel and a back channel made of a hydrogenated amorphous-silicon semiconductor layer sandwiched by thin silicon oxide insulating layers. The transfer characteristics are enhanced by uniformly shifting the threshold voltage to be higher than the maximum LC driving voltage (typically > 5 V). The enhanced TFT characteristics provided with a new driving scheme and shielding electrodes enables both the common line and black matrix to be eliminated. We fabricated an IPS TFT-LCD panel with aperture and contrast ratios that are 160% those of the conventional pixel structure.

## Introduction

After half a century of tremendous efforts in material research and device development and massive investments in advanced manufacturing technology, thin-film transistor liquid crystal displays (TFT LCDs) have now become the most common type of flat panel display^1^.

As the total worldwide LCD-related electrical energy consumption is significantly increasing due to the explosive increase in the use of displays for various purposes, especially for large-screen LCD TVs, the energy efficiency of TFT-LCDs should be improved to meet global sustainability requirements^2^. Here, while organic light emitting displays (OLEDs) prevail in small display markets such as for smartphones, tablets, and flexible displays, LCDs dominate in larger-screen TV applications because of their higher energy efficiency and ambient contrast ratio^3,4^. The high power efficiency of TFT-LCDs is due to their use of (1) an energy-efficient backlight unit, (2) an energy-efficient LCD panel, and (3) eco-friendly components^5^. The impact of their high energy efficiency is huge, amounting to an electricity savings potential of 32 terawatt hours [TWh] per year under the assumption that they replace inefficient analog TVs (mainly cathode ray tube type)^2^.

To meet energy-efficiency and image-quality requirements for TV applications, LCD panels with a high light-transmittance pixel structure have been developed by combining an LCD mode with an electrode structure enabling a high-aperture ratio in which light transmittance is electrically modulated^1^. In particular, the advent of the in-plane switching (IPS) LCD mode was ground-breaking as it afforded a very wide viewing angle for TV applications^6,7^, while the introduction of the fringe field switching (FFS) mode using LC materials with negative dielectric anisotropy dramatically improved the transmittance^8,9^. These LCD modes have been combined with a novel pixel structure with a noise-shielding electrode to increase the aperture ratio^10^. This pixel structure is called “IPS-Pro-Next” and was recently incorporated in a high-resolution 8K4K LCD with a diagonal size of 55 inches and with a backplane based on hydrogenated amorphous silicon (a-Si:H) TFT technology^11^.

In this paper, we describe a new way to increase the light transmittance of IPS TFT LCDs by combining a novel TFT switching mode with an electrode structure, potentially compatible with the FFS LCD mode. By introducing an enhanced switching mode of a-Si:H TFT wherein the threshold voltage is shifted higher than the maximum pixel voltage (typically > 5 V), we were able to eliminate the common line from the IPS TFT LCD panel by using the gate line as the common line and thereby increase the pixel aperture ratio. To enhance the transfer characteristics, we tried several methods and their combinations, such as using an MNOS (gate metal/SiN/SiOx/a-Si:H) vertical stacking structure^12^, gate bias stress and temperature application^13–17^, and back-channel oxidation^18,19^. We eventually found that an MNOS structure with back-channel oxidation but without gate-bias stress gave a uniform threshold voltage that was high enough to drive an active-matrix LCD. We fabricated a small IPS TFT-LCD panel combining this pixel structure with a noise-shielding electrode^9–11^ without using a black matrix layer and found that the displayed images were bright and had a high contrast ratio.

## Methods

**Figure 1:**
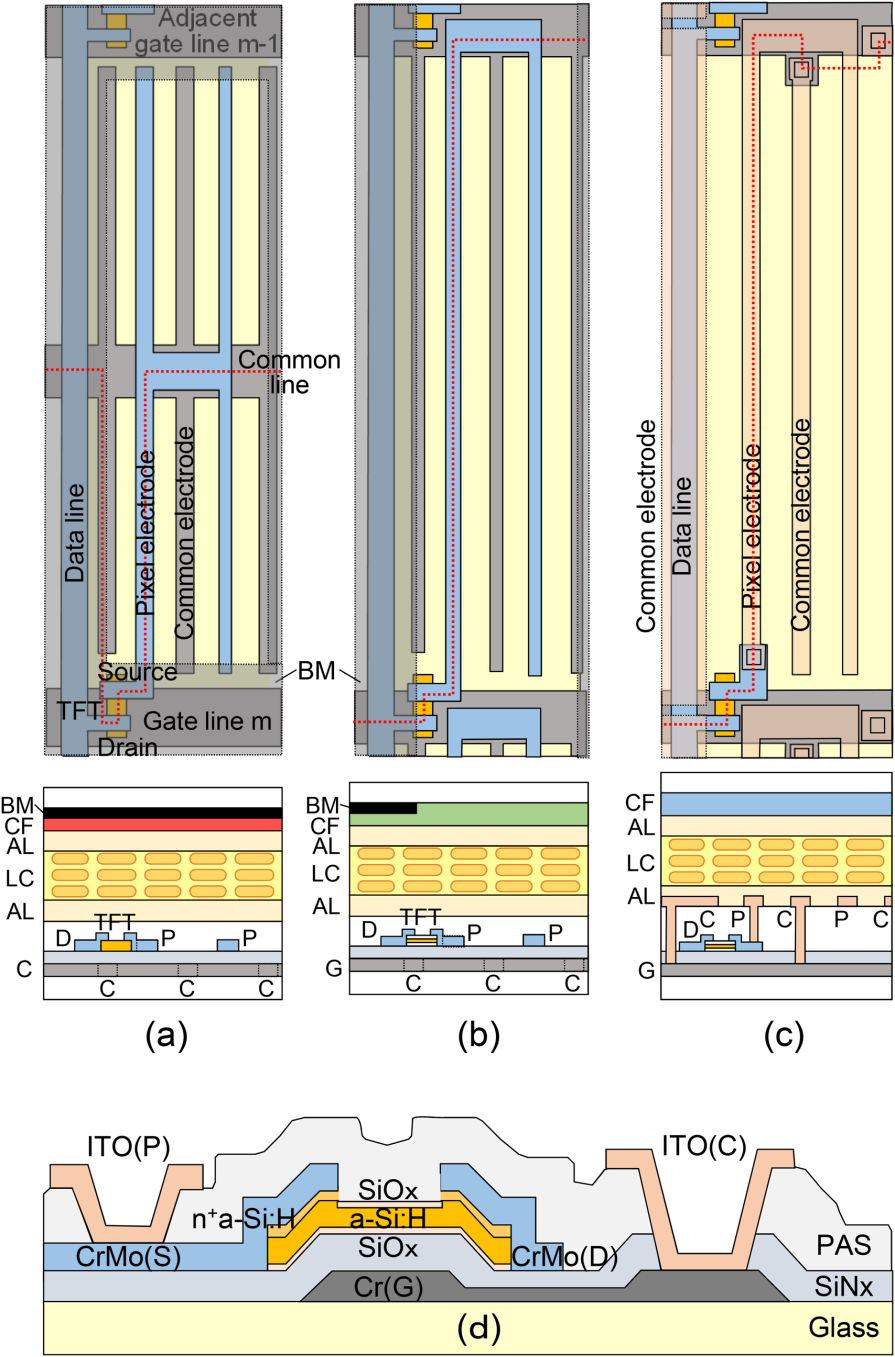
Pixel plane (top) and sectional views (bottom) of (**a**) the conventional structure, (**b**) the proposed CL structure without common line, (**c**) the proposed CLSE structure without common line and black matrix, with pixel and common electrodes made on the top of TFT substrate (G: gate, C: common, D: drain, P: pixel, AL: alignment layer, LC: liquid crystal, BM: black matrix, CF: color filter). Common electrodes are connected to the common line in the conventional structure, while they are connected to the adjacent gate line in the proposed structures. The red dotted line in each plane view indicates the cutting route for the corresponding sectional structure. (**d**) Detailed sectional structure of the CLSE structure.

Figure 1 shows pixel plane views and sectional views of three types of IPS TFT-LCD^20,21^ and the corresponding schematic sectional structures of the TFT substrates. Figure 1a shows the pixel plane (top) and sectional (bottom) views of the conventional structure, while Fig. 1b shows those of the structure we developed without a common line (“CL structure” in the following), which was simultaneously made with the conventional structure. Figure 1c shows those of the CL structure with pixel and common electrodes made by depositing ITO above the passivation layer. As the common electrode situated above the data line acts as a shielding electrode (SE) to prevent noise voltage from being applied to the LC, this structure is called a “CLSE structure” in the following.

In the IPS TFT-LCD, the comb (interdigital) electrodes, which apply an in-plane electric field to the LC layer, are composed of a pair of pixel and common electrodes, and the space between the pixel and common electrodes is transparent to incident light when pixel voltage is applied to the LC layer (normally off mode). In both the conventional and proposed pixel structure, the pixel electrodes are connected to the source electrode of the TFT. However, in contrast to the common electrodes in the conventional structure which are connected with a common line independently situated in parallel with a gate line, in the CL and CLSE structures they are connected with an adjacent gate line. Therefore, the proposed structures can eliminate the common line to increase the pixel aperture by using a gate line for supplying a common voltage as well as for addressing.

**Figure 2:**
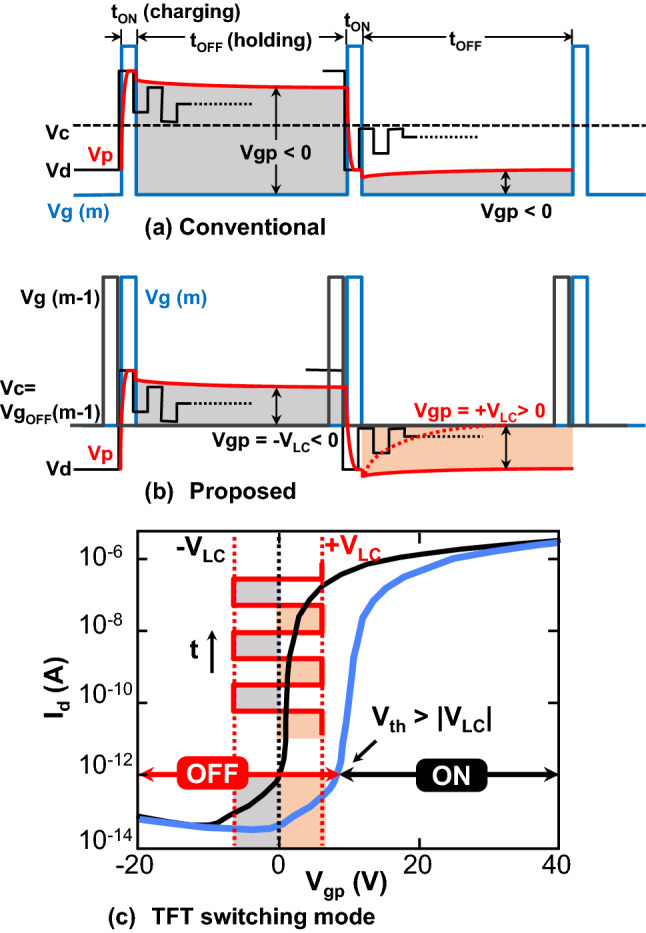
Driving waveforms of (**a**) conventional and (**b**) proposed pixel structures and (**c**) TFT transfer characteristics for conventional (black) and proposed (blue) structures with LC voltage, +/−V_LC_; the enhanced TFT characteristics with Vth larger than |V_LC_| are necessary to maintain the OFF state when a positive Vgp =  + V_LC_ is applied to TFT in the proposed pixel structures without the common line (Vg(m − 1): gate voltage of (m − 1)-th gate line, Vg(m): gate voltage of m-th gate line, Vd: drain voltage, Vc: common voltage, Vp: pixel voltage, Vgp: voltage between gate and pixel, t_ON_: duration of TFT ON state, t_OFF_: duration of TFT OFF state).

To remove the common line by using a gate line as a common line in the CL and CLSE structures shown in Fig. 1b,c, the transfer characteristics of the TFT must be enhanced such that the threshold voltage is higher than the maximum LC switching voltage, as explained using Fig. 2, in which schematic TFT-LCD driving waveforms are shown for (a) the conventional and (b) CL structures. In the TFT ON stage during t_ON_ with a high Vg (m), the LC capacitor is charged to supply Vd as a pixel voltage, Vp, and it is held in the TFT OFF stage during t_OFF_ with a low Vg (m). The drop in Vp at the transition from ON to OFF is called the feed-through voltage; the details are described in^22^. In the conventional structure with a common line, the common voltage (the dotted line in Fig. 2a), Vc, can be set independently as Vg and Vd, and it is possible to realize a condition in which the voltage difference between Vg and Vp (Vgp = Vgs = V_LC_) is always negative in the TFT OFF stage (the gray area in Fig. 2a). This is necessary to make the TFT OFF state since the threshold voltage of the conventional TFT is just above 0 V. On the other hand, in the CL structures, as the common voltage should be the same as the low voltage of the adjacent gate line m − 1, Vg (m − 1), Vgp becomes positive when Vp is lower than the low Vg value (the orange area in Fig. 2b). In this case, the conventional TFT enters the ON state and can not hold Vp, as shown by the red dotted curve. The relationship between the LC driving voltage, V_LC_ (= Vgp), and the TFT transfer (Id-Vg) characteristics is schematically shown in Fig. 2c. To maintain the OFF state in the CL structure, the TFT transfer characteristics should be enhanced such that the threshold voltage is higher than the maximum LC driving voltage, as shown by the blue transfer curve.

The benefits of the proposed pixel structure with the enhanced TFT are summarized here. The elimination of the common line not only increases the aperture area but also reduces the number of line crossings between the common and data lines, which should lead to an improvement in yield by reducing line short defects. In the conventional structure, the LC suffers from a continuous DC voltage during the holding (TFT OFF) stage even when the pixel voltage equal to zero, shown as the gray areas in Fig. 2a, which cause light leakage^23^ along the gate line through the gaps between the gate line and pixel/common electrodes. Therefore, in the conventional structure shown in Fig. 1a, a black matrix must be positioned above the gate line to prevent light leakage, which decreases the aperture. In contrast, in the CL structure with the enhanced TFT, the LC experiences zero volt in the dark state with Vgp = Vgc = 0; this allows the black matrix covering the gate line to be eliminated and thereby the aperture area to be increased, as will be experimentally shown later. Thus, the CL structure brings with it energy-efficiency benefits not only through the aperture increase but also through a yield increase.

The TFT fabrication process is explained by using Fig. 1d, which illustrates the schematic sectional structures of the TFT substrates. The conventional back-channel etched TFT structures were fabricated as follows^12,24^. After the gate line and common electrode patterning of the chromium metal sputtered on Corning 7059 substrates, silicon nitride, silicon oxide, intrinsic a-Si:H, and phosphorous doped n + a-Si:H films were successively deposited by using a PECVD system (Hitachi Kokusai Electric DL6010). The deposition temperatures of the SiOx/SiN gate insulator, channel-semiconductor, and doped-contact layers were 330, 280, and 230 °C, respectively. The thicknesses were 250 nm (SiN), 10 nm (SiOx), 200 nm (a-Si:H), and 50 nm (n + a-Si:H). The four consecutive layers were deposited in different chambers of a three-chamber PECVD system to avoid source gas contamination. In the CL structure shown in Fig. 1b, a passivation layer of 500-nm-thick SiNx (ε = 7) was formed to encapsulate the TFT except for the contact holes for the gate and data lines. TFT devices were fabricated using the conventional (five) photolithographic steps. Details of the process conditions are shown elsewhere^12^. In the CLSE structure shown in Fig. 1c, after a passivation layer of 3-μm-thick low-dielectric organic film (ε = 4) was formed, indium-tin-oxide (ITO) transparent pixel and common electrodes were formed on the passivation layer, and they were contacted with the electrodes underneath the passivation layer through contact holes. The details of the ITO deposition and patterning process are described elsewhere^25^. We used a dry-etching system for the BCO process an operated it under the following conditions: substrate temperature of 40℃, O2 gas pressure of 0.4 Torr, RF-power of 500 W, and gas flow rate of 500 sccm. The high-power condition is important to make a highly stoichiometric oxidized SiOx layer with an oxidation rate of 98% as determined from the SiOx ratio of the SiOx/a-Si:H mixture model used in spectroscopic ellipsometry (Jovin-Yvon UVISEL)^24^; the oxidation rate was calculated to be 3.7 nm/min.

The TFT transfer characteristics were enhanced in two ways. In the case of the MNOS TFT without back-channel oxidation, gate bias and temperature stress (BTS) were applied. In particular, after a gate bias of 80 V was applied for 10 s, the TFT was post annealed at 150 °C for 30 min in an N_2_ atmosphere for relaxation. In the case of the MNOS TFT with back-channel oxidation, the threshold voltage was high enough, so the BTS process was not applied. In the back-channel oxidation (BCO) process, the a-Si:H back-channel surface was oxidized in a plasma O_2_ treatment after the back-channel etching without breaking the vacuum in the same dry-etching system at room temperature. After the BCO, the TFT was post annealed at 200 °C for 1 h in an N_2_ atmosphere.

The contrast ratio (CR) was measured in a darkroom so that no ambient light induced reflections from the bus lines of the TFT substrate could cause a decrease in the CR. The IPS TFT-LCD panel used for the CR measurement was in normally off mode and the dark state was measured with the following applied voltages, Vgon = 30 V, Vgoff = 0 V, and Vd = 0 V. Other panel areas besides the CR measurement area were masked by a black sheet and the brightness was measured by using a photometer (Spectra Pritchard Photometer, Model 1980A).

## Results

**Figure 3:**
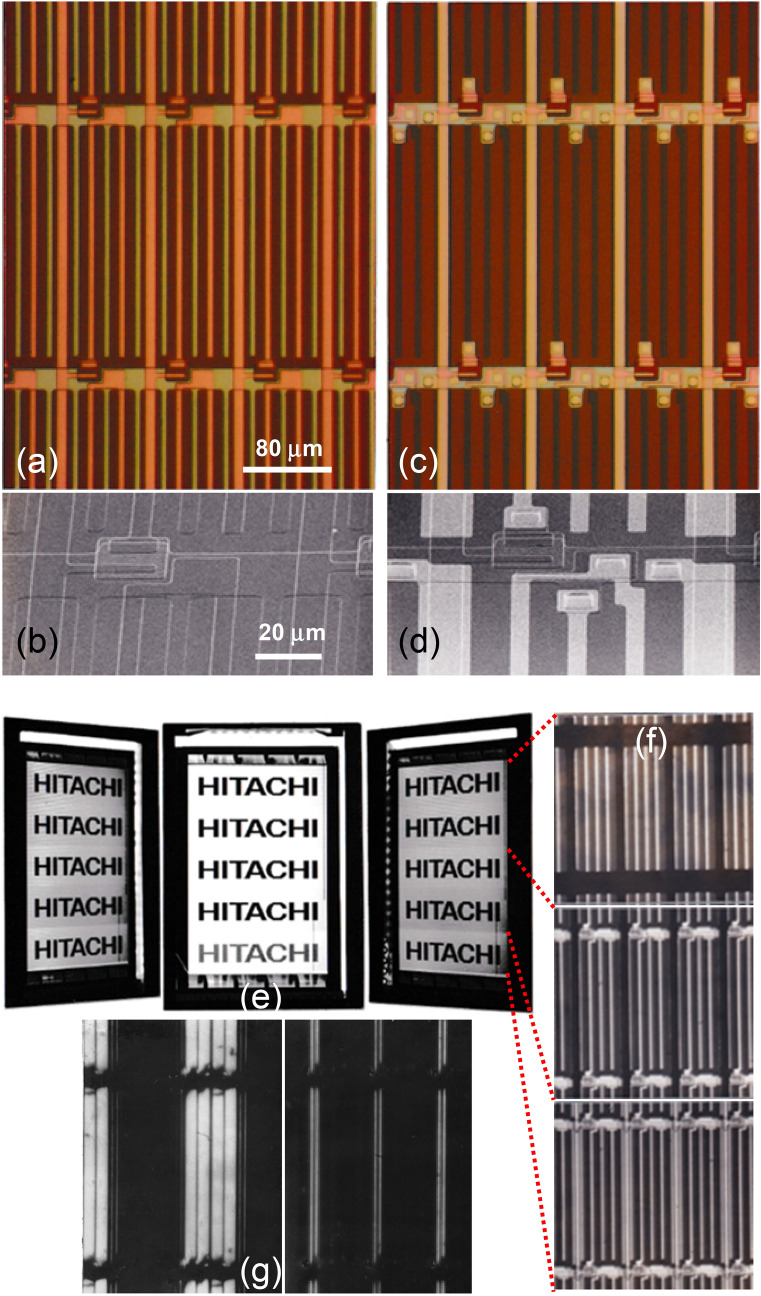
Optical and SEM (scanning electron microscopy) images of fabricated (**a**, **b**) CL and (**c**, **d**) CLSE pixel structures. The five white line patterns in (**d**) are the ITO interdigitated pixel and common electrodes. (**e**) Images from the normal direction and from 50 degrees to the left and right of a 2.3-inch-diagonal display incorporating the IPS TFT-LCD panel. (**f**) The three-black matrix (BM) patterns (top: BM covering both gate and data lines, middle: BM covering only the data lines, and bottom: without BM) and (**g**) optical images of pixels without BM (left: LC on and off voltages supplied to every other data line, right: LC off voltage supplied to all data lines).

Figures 3a–d show optical and SEM (scanning electron microscopy) images of the fabricated CL and CLSE pixel structures. Each structure has the same pixel size (80 μm × 240 μm) and minimum pattern size (5 μm). As shown in Fig. 3d, the white line patterns are the ITO interdigitated pixel and common electrodes. They are well connected to the source electrode and gate line via through holes, and the common electrodes at both ends cover the underlying data lines to prevent electrical noise from being applied to the LC layer. As will be shown later, this noise shield electrode (SE) is what makes the black matrix above the data line unnecessary^9–11,23^.

Figure 3e shows images from the normal direction and from 50 degrees to the left and right of a 2.3-inch-diagonal display incorporating the IPS TFT-LCD panel fabricated in our laboratory, (f) the three black matrix (BM) patterns (top: BM covering both gate and data lines, middle: BM covering only the data lines, and bottom: without BM), and (g) optical images of panel areas without the BM (left: LC on and off voltages supplied to every other data line, right: LC off voltage supplied to all data lines). As can be seen in the image from the normal direction, the brightness and contrast of the display area with the top BM and middle BM patterns are almost the same, but the contrast of the display area without the BM is relatively lower because of the lower darkness level of the LC off pixels indicating “HITACHI”. As shown in Fig. 3g, this is due to light leaking through the aperture between the data line and adjacent common lines. Therefore, in the CL structure, the BM on the drain line is necessary to obtain a high contrast ratio by shielding light leakage. This is the same as in the conventional structure. On the contrary, there is no light leakage along the gate line through the gaps between the gate line and edges of the pixel/common electrodes, as is clearly shown in Fig. 3g. This is a unique advantage of the CL structure because the conventional structure must shield these gaps with the BM to prevent light leakage. The suppression of light leakage along the gate line in the CL structure is due to the driving scheme (see Fig. 2b,a for a comparison with the conventional structure). During the holding period (t_OFF_) in the conventional structure, regardless of the pixel voltage, Vp (including Vp = 0), nonzero Vgp and Vgc are always applied to keep the TFT off, and these voltages are applied to the LC layer, inducing light leakage as reported in^23^. On the other hand, in the case of the dark state with Vp = 0 in the CL structure, both Vgp and Vgc are zero volt, which does not induce light leakage. It is possible for the CL structure to maintain the off state with Vgp = Vgc = 0 V because of the enhanced TFT characteristics. Thus, the CL structure can increase the aperture ratio because of the elimination of the common line and the BM along the gate line.

**Figure 4:**
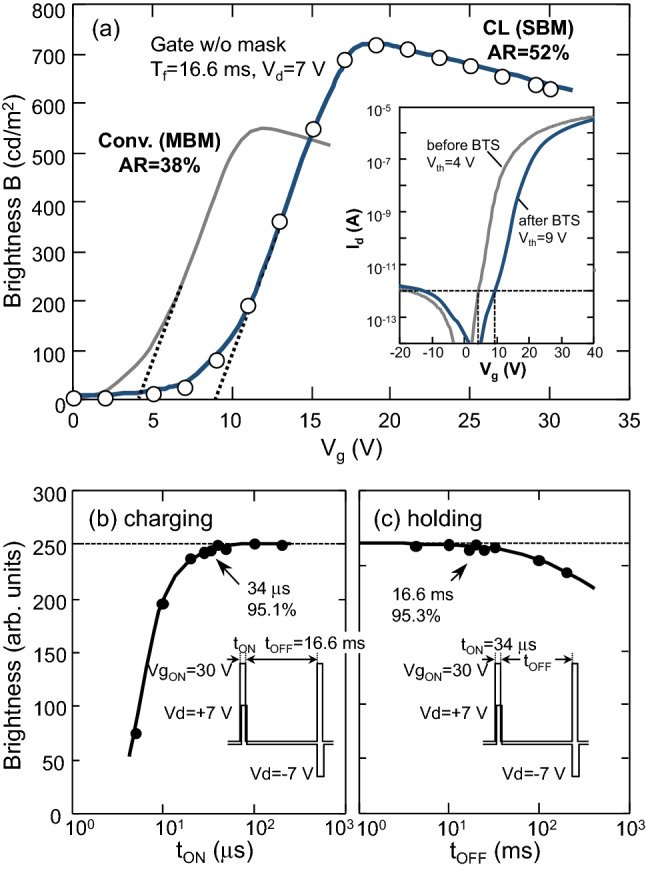
(**a**) Dependence of panel brightness and TFT current on gate voltage for the conventional pixel structure of 38% aperture ratio (AR) with matrix black matrix (MBM) over both drain and gate lines and proposed CL pixel structure of 52% AR with strip black matrix (SBM) over only the drain lines. Transfer characteristics before and after bias temperature stress (BTS) treatment are shown in the inset. (**b**) Charging and (**c**) holding characteristics of enhanced TFT of the CL structure with gate as a common line. Vg and Vd in the TFT ON state are 30 V and +/−7 V, respectively.

Figure 4a shows the gate voltage (Vg) dependence of the panel brightness, while the inset shows that of the TFT current (transfer characteristics). The gray curves are for the conventional IPS TFT-LCD with the TFT before enhancement, the common line, and the matrix BM (MBM) shown at the top of Fig. 3f. The blue curves are for the proposed CL structure with the enhanced TFT and the stripe BM (SBM) shown in the middle of Fig. 3f. In this case, enhanced TFT characteristics were obtained by using an MNOS TFT without back-channel oxidation that was enhanced by the BTS process. In both structures, the threshold voltages for panel brightness, defined by extrapolating the straight part of the brightness curves, reflect those of the TFT transfer curves defined as Vg at a drain current of 10^−12^ A, and they are well matched to be 4 V and 9 V, respectively. The maximum brightness for the CL structure is 137% higher than that for the conventional structure, which is due to the increase in the aperture ratio from 38 to 52% that results from the elimination of the common line and the BM covering the gate line.

To estimate the charging and holding characteristics of the MNOS-enhanced TFT in the panel, the gate TFT ON and OFF time dependences of the panel brightness were measured (Fig. 4b,c). The charging characteristics in Fig. 4b are plotted as a function of t_ON_ at t_OFF_ = 16.6 ms, Vg = 30 V, and Vd = ± 7 V. The holding characteristics in Fig. 4c are plotted as a function of t_OFF_ at t_ON_ = 34 μs. 95.1% charging at t_ON_ = 34 μs and 95.3% holding at t_OFF_ = 16.6 ms indicate that the enhanced TFT has sufficient charging and holding performance to drive a standard VGA (640 × 480 pixels) panel (the number of scanning lines is estimated as t_OFF_/t_ON_ = 16.6/0.034 = 488).

**Figure 5:**
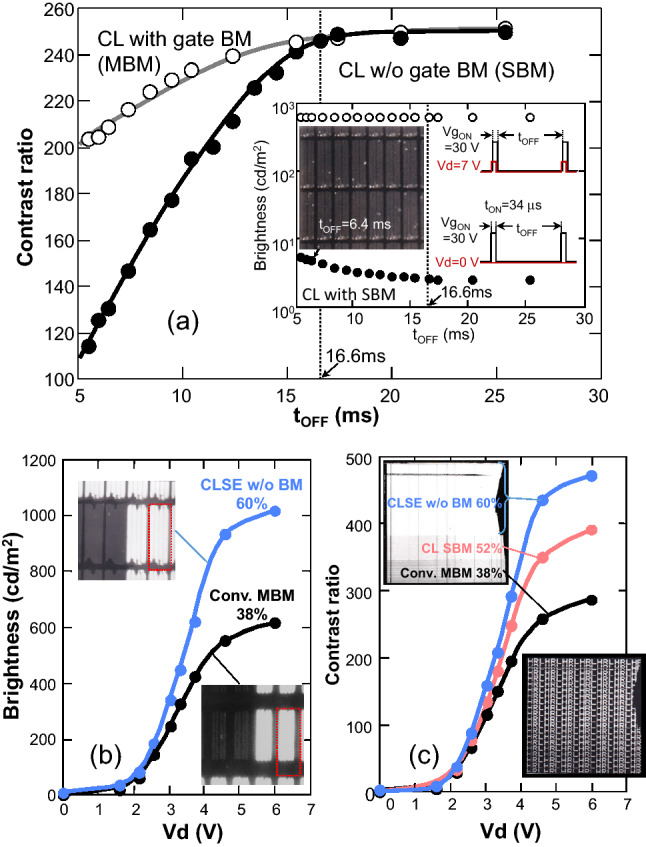
(**a**) Contrast ratio (CR) for CL panels with matrix BM (MBM) and stripe BM (SBM) plotted as a function of t_OFF_. The inset shows the brightness in the bright (Vd = 7 V) and dark (Vd = 0 V) states of the CL panel with the SBM as a function of t_OFF_ and an optical image of the panel with t_OFF_ = 6.4 ms. Vd (= V_LC_) dependence of (**b**) brightness and (**c**) contrast ratio of the CLSE panel. Insets of (**b**): optical images indicating the elimination of the BM from the CLSE structure (upper left) and the MBM in the conventional structure (lower right). Insets of (**c**): CLSE panel composed of different areas with three different pixel structures and aperture ratios (ARs), i.e., CLSE structure without BM (60%), CL structure with SBM (52%), and conventional structure with MBM (38%).

To confirm the driving conditions for the CL structure without the BM along the gate line (with the SBM), the t_OFF_ dependence of the contrast ratio (CR) in the CL panel was further investigated as shown in Fig. 5a, where CR is plotted as a function of t_OFF_ for the CL panels with the matrix BM (MBM) and the strip BM (SBM). The inset shows the brightness in the bright (Vd = 7 V) and dark (Vd = 0 V) states of the CL panel with the SBM as a function of t_OFF_ and an optical image of the panel with t_OFF_ = 6.4 ms. The SBM and MBM panels keep CR higher than 240 with t_OFF_ > 16.6 ms, the frame period of a display panel without flicker being noticeable to the human eye. both panels decrease CR when t_OFF_ is less than 16.6 ms; the CR of the SBM panel decreases faster than the CR of the MBM panel. As shown in the inset, the decrease in CR was due to the increase in dark-state brightness with decreasing t_OFF_ as light leakage increases along the gate line. This light leakage is induced by the voltage Vgp = Vgc = Vg_ON_ = 30 V applied only for 34 μs during the TFT ON (charging) state, which is 1/488^th^ the duration, t_OFF_ = 16.6 ms, of the TFT OFF (holding) state with Vgp = Vgc = Vg_OFF_ = 0 V, but the ratio increases with decreasing t_OFF_ and becomes effective enough to switch on LC layer and induce light leakage. However, it should be stressed again that the CL panel with the normal holding (TFT OFF) time of 16.6 ms does not suffer from the light leakage along the gate line, so the aperture ratio can be increased by removing the BM along the gate line.

Figure 5b,c shows the Vd (= V_LC_) dependence of the brightness and contrast ratio of the CLSE panel without a BM along the drain line and along the gate line. As shown in the optical images in the insets of Fig. 5c, the CLSE panel has different areas with three different pixel structures and aperture ratios (ARs), i.e., the CLSE structure without the BM (60%), the CL structure with the SBM (52%), and the conventional structure with the MBM (38%). The optical images in the inset of Fig. 5b clearly indicate the elimination of the BM from the CLSE structure and the MBM in the conventional structure. The ratios of the bright area in the CLSE and conventional pixels shown in the red dotted square appear higher than the aperture ratios because the pixel and common electrodes are invisible due to the brightness. The brightness and contrast ratio in each area increase with increasing Vd; the ratios for the CLSE structure without the BM are approximately 160% those of the conventional structure with the MBM, reflecting the difference in aperture ratio.

Below, we investigate the process for achieving both a high and uniform threshold voltage to drive the CL and CLSE IPS-LCD panels.

**Figure 6:**
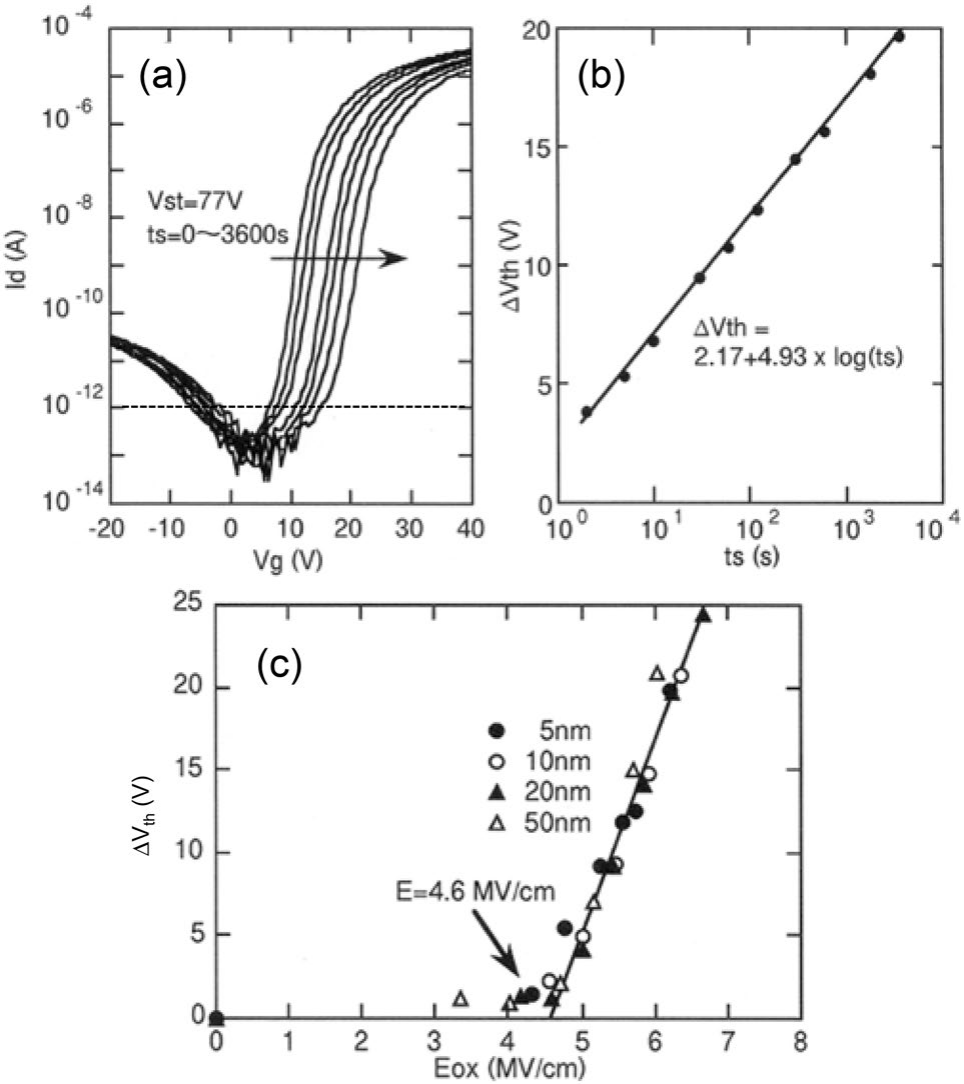
Effect of bias temperature stress (BTS) on TFT characteristics. (**a**) The transfer (Id–Vg) curve shifts in the positive direction with positive gate bias stress of Vst =  + 77 V over the duration of 0–3600 s. (**b**) ΔVth, defined as the Vth shift from the initial value via BTS, increases logarithmically with increasing t_S_, following ΔVth = 2.17 + 4.93 × log (t_S_). (**c**) ΔVth of MNOS TFTs with different SiOx thicknesses from 5 to 50 nm as a function of the electric field applied to the SiOx layer.

Figure 6 indicates the effect of bias temperature stress (BTS) on the TFT characteristics. As the stressing time, t_S_, of the positive gate stress voltage, Vst =  + 77 V, increases from 0 to 3600 s, the transfer (Id-Vg) curve shifts in the positive direction (Fig. 6a). Vth is defined as Vg at which Id = 10^−12^ A and ΔVth is defined as the Vth shift from the initial value via BTS. As shown in Fig. 6b, ΔVth increases logarithmically with increasing t_S_: ΔVth = 2.17 + 4.93 × log (t_S_). The mechanism behind the gate-stress-induced Vth shift is electron tunnel injection from the a-Si:H semiconductor into the SiOx gate insulator. For confirmation, ΔVth of MNOS TFTs with different SiOx thicknesses is plotted as a function of the electric field applied to the SiOx layer in Fig. 6c. Here, the thickness of the SiOx was varied (5, 10, 20, 50 nm), while the SiN thickness was fixed at 200 nm. The electric field applied to SiOx, E_ox_, was calculated using the following equation,

1 $${E}_{ox}= \frac{{\varepsilon }_{n}}{{\varepsilon }_{o}\cdot {d}_{n}+ {\varepsilon }_{n}\cdot {d}_{o}} \times \frac{{V}_{st}}{{d}_{o}}$$

where ε_o_ = 3.5 and d_o_ are the electrical permittivity and thicknesses of SiOx and ε_n_ = 7 and d_n_ = 200 nm are those of SiN. ΔVth linearly increases at a threshold electric field at 4.6 MV/cm on the same straight line regardless of the thickness of SiOx. From these results, we concluded that the Vst-induced Vth shift mechanism is Fowler–Nordheim type tunneling injection^26^ of electrons through the SiOx.

**Figure 7:**
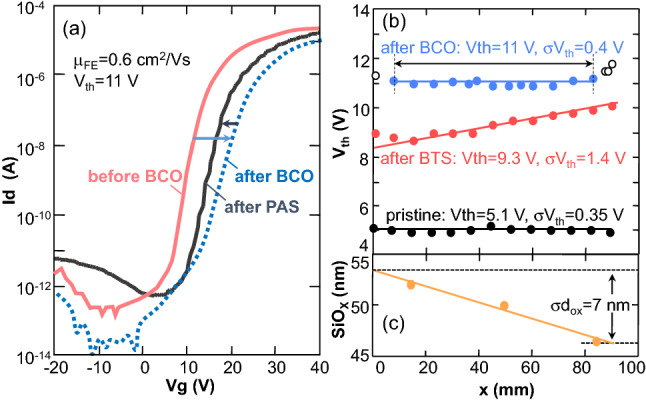
(**a**) Effect of back-channel oxidation (BCO) and passivation (PAS) on Id–Vg characteristics of MNOS TFT. (**b**) Vth and Vth standard deviation, σVth, after BTS and after BCO without BTS treatments and (**c**) SiO_X_ thickness as a function of position along gate-line, x.

Figure 7a shows the effect of back-channel oxidation (BCO) and passivation (PAS) on the Id-Vg characteristics of the MNOS TFT. The Id-Vg curve with Vth = 5.1 V is further enhanced to Vth = 10.9 V after BCO, although there is a slight degradation of the slope of the current increase in the sub-threshold region. The slope recovers after PAS without any change to the enhanced characteristics^24^.

The advantage of BCO over BTS is the uniformity of the enhanced characteristics; Fig. 7b shows the distribution of the enhanced Vth as a function of position along gate-line, x, for the MNOS TFTs connected with a 90-mm-long gate line in the TFT substrate after BTS and BCO treatments. The average value of Vth and the standard deviation, σVth, for pristine TFTs before BTS or BCO, are 5.1 V and 0.35 V, respectively. After BCO, Vth is uniformly enhanced, with an average Vth = 11 V and σVth = 0.4 V without increasing σVth. On the other hand, after BTS, Vth is nonuniformly enhanced with an average Vth = 9.3 V and σVth of 1.4 V. In particular, Vth increases linearly as a function of position along gate-line, x. As the gate SiOx thickness linearly decreases, from (54 nm) to (47 nm) with increasing x as shown in Fig. 7c, the increase in ΔVth after BTS with x is due to increase in the electric field applied to SiOx as shown in Fig. 6c and Eq. 1.

**Figure 8:**
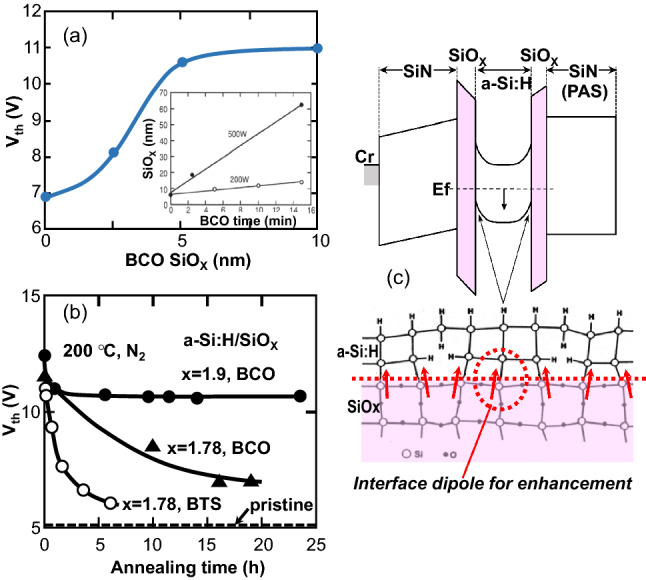
(**a**) Dependence of Vth on thickness of back-channel oxidized (BCO) SiO_X_. The inset shows the SiOx thickness dependence on the BCO process time for RF powers of 200 W and 500 W. (**b**) Stability of Vth as a function of annealing time for three different TFTs with gate SiOx stoichiometry, x = 1.78 and bias temperature stress (BTS), x = 1.78 and BCO, and x = 1.9 and BCO. The temperature of annealing in the N_2_ atmosphere was 200 °C. (**c**) Band diagram of back-channel oxidized MNOS TFT. The red arrows show the electric dipoles at the SiO_X_/a-Si:H and a-Si:H/BCO SiO_X_ interfaces.

It has been shown that the Vth of the MNOS TFT is independent of the gate SiOx thickness when the thickness is more than 5 nm^12^, a benefit for uniformly enhanced TFT characteristics. As shown in Fig. 8a, Vth of the MNOS TFT with the BCO treatment becomes almost independent of the BCO SiOx thickness. As shown in the inset, the thickness of BCO SiOx composed of oxidized a-Si:H linearly increases with BCO processing time, and the Vth increase almost saturates at a BCO SiOx thickness greater than 5 nm. Therefore, the enhanced MNOS TFT after BCO has a uniformly high Vth that is robust to thickness fluctuations of the gate and BCO SiOx layers. BCO also has an advantage over BTS in terms of the stability of Vth as shown in Fig. 8b, which plots the annealing time dependence of Vth for BCO and BTS. In this experiment, the stoichiometry (x) of the gate SiOx was 1.78 for BTS and 1.78 and 1.9 for BCO. The annealing temperature in the N_2_ atmosphere was 200 °C. In the case of BTS, Vth decreased to the pristine value after approximately 5 h of annealing, while Vth decreased more slowly in the case of BCO. In particular, the annealing time required for Vth to fall to 7 V was 7.5 times longer than that of BTS. Increasing the stoichiometry (x) of the gate SiOx dramatically improved the BCO-enhanced Vth to as much as 11 V, which was stably maintained after 24 h of annealing at 200 °C.

A schematic band model for the MNOS TFT with the BCO treatment and the sectional structure of the interface between the a-Si:H and gate and BCO SiOx are shown in Fig. 8c. The uniformly high Vth that is independent of SiOx thickness is due to the dipoles generated at the channel and back-channel interfaces between a-Si:H and SiOx^12^. The dipoles cooperatively bend the a-Si:H band downward to prevent electrons from accumulating at the a-Si:H/gate SiOx interface, which results in high Vth characteristics. It should be stressed that the key to achieving the high uniformity of the high threshold voltage is not the uniformity of the back-channel oxidized SiOx thickness but rather the uniformity of the strength of the dipoles at the back-channel interface that is independent of the thickness. To get a uniform dipole strength, the oxidized SiOx layer should have high stoichiometry as in the CVD-deposited SiOx layer shown in Fig. 8 of reference^12^.

## Discussion

The enhanced TFT driven active-matrix LCD was developed to increase the aperture ratio of IPS-LCDs. It has a threshold voltage higher than the maximum LC driving voltage, which enables the gate line to be used as a common line without degrading the LCD driving performance. This allows the common line to be removed, which increases the pixel aperture ratio (i.e., the CL structure). Furthermore, it frees the LC from the effect of the DC voltage applied between the gate line and pixel/common electrode and the light leakage through the gaps between the gate line and the pixel/common electrodes caused by the DC voltage. Therefore, the BM covering the gate line becomes unnecessary, and the aperture ratio of the CL structure can further be increased. By combining the CL structure with an electrical noise shielding electrode covering the drain line (i.e., the CLSE structure), the whole BM can be removed without affecting the image quality, which further increases the aperture area. Regarding the practicality of a future IPS TFT-LCD panel without a BM, there are mainly four roles for the BM: (1) as a shield against light leaking from pixels when the color black is displayed, (2) as a shield against light coming from the adjacent pixels to prevent color mixing, (3) as a shield against reflections of ambient light from the metal bus lines in the TFT substrate, and (4) as a shield against light irradiating the TFT channel to prevent photo-current in the TFT. The CLSE pixel structure without a BM has no light leakage along the gate and drain lines, as shown in the optical image in Fig. 5a; this means role (1) becomes unnecessary. It is difficult, however, for the CLSE structure alone to make (2), (3), and (4) unnecessary; it should be combined with other technologies such as a color filter on TFT (COT)^19,27^ or black photo spacer (BPS)^28,29^. The COT structure moves the color filter (CF) and black matrix (BM) from the upper substrate to the TFT substrate. As the distance between the CF/BM and the pixel decreases from about 3 μm to 0 μm, the optical crosstalk between adjacent pixels is suppressed enough to make (2) unnecessary. The BM is still necessary for (3) and (4), but the complexity of its fabrication process can be reduced by making it with the spacer at the same time in the BPS process^28,29^.

There is a report on replacing the BM with two adjacent stacked CF layers with reduced transmittance covering the drain/gate lines and the channel of the TFT as a way to shield from ambient light^30^.

As the shielding electrode configuration to reduce the BM along the drain line has been reported^9,11^, the distinctive advantage for the CL and CLSE structure is to reduce or remove the BM along the gate line due to the unique driving voltage scheme enabled with the enhanced TFT characteristics.

To enhance the TFT characteristics, we developed a new TFT with a vertical stacking structure (metal/SiN/SiOx/a-Si:H/SiOx/passivation) in which the channel and back channel consists of a a-Si:H semiconductor layer sandwiched with SiOx. The gate SiOx and back-channel SiOx are made using PE-CVD and the back-channel oxidation (BCO) process. The highly stoichiometric gate SiOx and BCO back-channel SiOx generate electric dipoles at both interfaces and provide a uniform and stable Vth higher than the maximum LC driving voltage. Enhanced TFT driven LPS-LCDs were fabricated and our experiments on them showed that the aperture and contrast ratios were up to 160% those of the conventional structure. The uniformity and operational stability of the TFT characteristics need to be further studied before the CL and CLSE structures can be incorporated in large, high-resolution IPS-LCD panels.

The TFT threshold voltage, i.e., the gate voltage applied to make the channel conduct electricity, is especially important for display applications^31^. Variability between individual devices, i.e., the standard deviation, σVth, is an important metric of the brightness of the individual pixels and reflects the luminance variation across the display. In this sense, our enhancement in Vth of a-Si:H TFTs by using high stoichiometric gate SiOx and BCO can give a high and uniform Vth to drive the CL and CLSE pixel structure for IPS-LCDs. Here, a previous study reported an enhanced TFT for driving an active-matrix LCD^32^. The purpose was not to increase the aperture ratio but to improve the fabrication process by doping to obtain a uniform Vth, but it was not larger than the maximum LC driving voltage^33^.

Organic TFTs are promising for future large flexible displays made by energy-efficient printing processes^34^, and they can drive active-matrix LCD panels^35^. For enhanced organic TFTs, the BTS process cannot obtain a stably high Vth because of the Vth shift which occurs through reversible electron trapping in semiconductor molecules^36,37^. A promising method for obtaining a stably high Vth is to insert a self-assembled monolayer (SAM) between the gate insulator and organic semiconductor, since the dipole of the chemically fixed SAM at the interface uniformly enhances the transfer characteristics and Vth can be controlled through appropriate dipole design of the SAM^38^.

IPS TFT-LCD technologies are expected to continuously progress in terms of image quality and energy efficiency through improvements in TFT performance, electrode design, and materials, such as a recently proposed material that can be inserted between the pixel electrodes and the LC to realize high transmittance and a fast response^39,40^.

## Conclusions

We developed active-matrix in-plane switching liquid crystal displays (IPS-LCDs) with a new stacking structure of thin-film transistors (TFTs) with an aperture ratio of 60%. Because of the electrical dipoles of the channel and back-channel interfaces of the gate metal/SiN/SiOx/a-Si:H/SiOx TFT structure, the TFT transfer curve is uniformly enhanced such that Vth is higher than the maximum LC driving voltage (typically > 5 V). The improved TFT characteristics enabled the common line to be eliminated and the adjacent gate line to be used as the common line (CL pixel structure) without degrading the image quality of the IPS-LCD. The CL structure also eliminates the need for continuously applying a DC TFT switching off voltage to the LC between the gate and common lines in the conventional pixel structure and eliminates the need for positioning a conventional BM over the gate line. Further, the CL structure combined with shielding electrodes over the data line (CLSE pixel structure) completely eliminates the need for a BM. Experiments conducted on a fabricated IPS TFT-LCD panel indicated that the aperture and contrast ratios are 160% those of the conventional structure.
